# Plasmapheresis for Pembrolizumab-Induced Hepatitis in a Patient with Squamous Cell Carcinoma and Prior Orthotopic Liver Transplantation

**DOI:** 10.1155/2022/5908411

**Published:** 2022-01-21

**Authors:** Sanaa Al-Nattah, Kusum Lata Sharma, Matthew Caldis, Erin Spengler, William N. Rose

**Affiliations:** ^1^Department of Pathology and Laboratory Medicine, University of Wisconsin Hospital, 600 Highland Ave, Madison, WI 53792, USA; ^2^Department of Medicine, University of Wisconsin Hospital, 600 Highland Ave, Madison, WI 53792, USA

## Abstract

Checkpoint inhibitor therapy with monoclonal antibodies against programmed cell death protein 1 (PD1) has been implemented in the treatment of numerous malignancies. Pembrolizumab is one such medication. While severe toxicities are very rare, mild immune-mediated reactions with a variety of end organ injuries are among the most commonly encountered adverse events attributed to these medications. Acute liver injury manifesting as biochemical abnormalities with or without synthetic dysfunction is one such potential adverse reaction. Rarely, a relatively severe hepatitis can occur. While such severe adverse events are often successfully managed with systemic corticosteroids and drug discontinuation, our patient was refractory to standard management. We present a case of pembrolizumab-induced hepatitis in a patient with squamous cell carcinoma and prior orthotopic liver transplantation. Through a combination of serial plasmapheresis and intravenous immunoglobulin (IVIG) infusion, the patient's hepatitis resolved as evidenced by virtual normalization of his liver indices. This illustrates the effectiveness of a relatively novel treatment strategy for this rare side effect of checkpoint inhibitor antineoplastic therapy.

## 1. Case Presentation

A 76-year-old Caucasian man with end-stage liver disease secondary to alcoholic cirrhosis underwent orthotopic liver transplantation and was maintained on low-dose immunosuppression of tacrolimus monotherapy with good graft function. His course was complicated by stage III metastatic laryngeal squamous cell carcinoma (T2, N1, and M0) with a positive lymph node on the left side of the neck with negative laryngoscopy.

He underwent three cycles of chemotherapy consisting of weekly cisplatin followed by radiotherapy with reduction in the dose of tacrolimus to minimize side effects. Following radiation therapy, he developed severe dysphasia and aspiration requiring feeding tube placement.

Nine months after the completion of radiotherapy, he had a recurrence of squamous cell carcinoma with lung metastasis. The metastasis was diagnosed in a painful left supraclavicular lymph node that required a left-sided neck dissection. As a result of this metastasis, he received pembrolizumab (Keytruda). His tacrolimus dose was decreased, and prednisone was added at 5 mg daily.

Pretreatment liver function values (LFT's) included serum alanine aminotransferase (ALT) 24 U/L, aspartate amino transferase (AST) 16 U/L, alkaline phosphatase (ALP) 108 U/L, gamma glutamyl transferase (GGT) 17 U/L, and bilirubin of 0.2 mg/dl.

Repeat lab test results after 2 weeks of the first dose of pembrolizumab were within normal limits. However, repeat lab tests after 3 weeks were abnormal with ALT 783 U/L, AST 413 U/L, ALP 444 U/L, and bilirubin 2.1 mg/dl.

At this time, his tacrolimus trough was <2, and the only clinical symptom was jaundice. An abdominal ultrasound ruled out biliary obstruction and portal vein thrombosis.

Viral serologic testing for cytomegalovirus (CMV), Epstein–Barr virus (EBV), BK virus (BKV), hepatitis viruses, herpes simplex virus (HSV), varicella zoster virus (VZV), human herpes virus (HHV), and respiratory syncytial virus/severe acute respiratory syndrome coronavirus 2 (RSV/SARS-CoV-2) were negative.

Prednisone was increased to 60 mg PO daily and then to 100 mg daily without improvement in his liver function.

**Figure 1:**
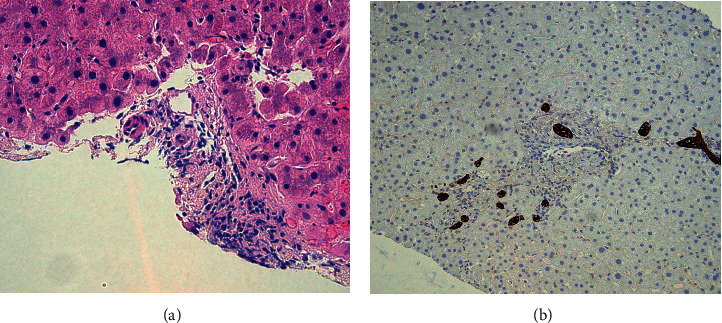
(a) A representative field from the H&E stained liver biopsy shows duct dropout, a lack of prominent mixed inflammatory infiltrate, and a portal tract with endotheliitis. (b) A representative field from the CK7 stained liver biopsy highlights bile ductular reaction. Not shown: a trichrome stain showed stage I fibrosis.

He was admitted to the hospital and treated with 80 mg IV methylprednisolone with minimal improvement. Due to minimal steroid response, mycophenolate 500 mg BID, thymoglobulin 1.5 mg/kg, and ursodiol 300 mg BID were given. A liver biopsy revealed portal venous and capillary endothelialitis and complete duct dropout without a classic portal mixed inflammatory infiltrate indicative of cellular rejection (Figures 1(a) and 1(b)).

Severe immune-mediated hepatitis with vanishing bile duct syndrome and cholangiopathy was diagnosed. Given the clinical history of dramatic elevation in alkaline phosphatase since starting treatment with pembrolizumab instead of a gradually progressive elevation of alkaline phosphatase, drug-induced immune mediated injury was favored over ductopenic rejection. In other words, given the strong temporal relation to pembrolizumab therapy and laboratory trends, a drug-induced immune mediated injury was felt to be the most likely etiology in our patient. A RUCAM score of 9, which indicates a high probability that the liver injury was due to pembrolizumab, was calculated.

Liver function tests were still not improving. Thus, IV dexamethasone 50 mg and antithymocyte immunoglobulin 1.5 mg/kg per day were added. Tacrolimus was increased to 2 mg BID with a goal trough of 6–8. Mycophenolate was increased to 1000 mg BID. A magnetic resonance colangiopancreatography (MRCP) revealed mild anastomotic narrowing of the common bile duct (CBD). Given the histologic diagnosis of immune-mediated hepatitis, this mild narrowing was not considered to be the primary cause of the histologic changes.

His LFTs started to improve. Thymocyte globulin was withheld but was restarted again after declining function was observed again. In sum, despite treatment with a variety of immunosuppressive agents including high-dose IV corticosteroids, antithymocyte globulin, mycophenolate, and increased doses of tacrolimus in addition to ursodiol, his liver failed to show significant improvement. Thus, his liver injury appeared to be refractory to medical immunosuppression.

Given the IgG nature of pembrolizumab, we started plasmapheresis for drug removal and IVIG for immunomodulation. Each centrifugal plasmapheresis was a 1-volume exchange that used 5% albumin as the replacement fluid. Daily plasmapheresis was initially performed on four consecutive days. Of note, the third plasmapheresis lasted only 15 minutes and was terminated due to shoulder pain and hypotension. An infusion of 100 m/kg IVIG was added after each of these four initial plasmapheresis procedures.

Before these four initial plasmapheresis procedures, LFT's were AST 87 U/L, ALT 222 U/L, ALP 469 U/L, and bilirubin 15.3 mg/dl. Following these treatments, his liver function improved with LFT's, AST 60 U/L, ALT 82 U/L, ALP 143 U/L, and bilirubin 10.3 mg/dl.

After receiving additional two plasmapheresis procedures with IVIG, he was discharged from the hospital. Total number of inpatient plasmapheresis with IVIG was 6 (this includes 1 incomplete). As an outpatient, he received both plasmapheresis and IVIG twice a week for 4 more weeks for a total of 8 outpatient procedures.

After a total of 14 inpatient and outpatient treatments, his procedures were discontinued, and liver function values were monitored continuously while he was on tacrolimus, mycophenolate, and a prednisone taper. Currently, he is receiving non-immune-mediated chemotherapy with carboplatin and docetaxel for his lung metastasis. His most recent LFT's were AST 19 U/L, ALT 16 U/L, ALP 147 U/L, and bilirubin of 0.5 mg/dl.

## 2. Discussion

The programmed cell death protein 1 (PD1) is a checkpoint that regulates the immune response. Ligation of PD1 with its ligands PDL1 and PDL2 results in transduction of negative signals to T-cells. The expression of PD1 on effector T-cells and PDL1 on neoplastic cells allows tumor to evade the immune response. Blockade of PD1 is an important immunotherapeutic strategy for cancers. Pembrolizumab (Keytruda) is a humanized monoclonal anti-PD1 antibody that has been extensively investigated in numerous malignancies [1].

Though adverse events occurred in up to 60% of patients, grade 3/4 toxicities were observed in <10% of cases. Besides infusion reactions, most of the AEs are thought to be immune-related AEs, occurring in any organ. Since the optimal duration of treatment is undefined, pembrolizumab may be given for up to 2 years. Consequently, immune-related AEs can occur late or even after cessation of treatment [1].

One of the known side effects of checkpoint inhibitor immunotherapy is the onset of mild liver biochemical abnormalities at 3–6 months after starting treatment is [2]. It generally manifests as a mixed pattern of liver function test abnormalities.

Multiple patterns of histological injury have been documented in the literature [3]. In a study of 491 consecutive patients receiving pembrolizumab, liver injury occurred in 70 (14%) patients. Generally, liver injury was predominantly cholestatic and mild at onset with similar baseline demographic and laboratory features of the patients with liver injury versus those without liver injury. Only a minority of the liver injury cases were attributed to pembrolizumab hepatotoxicity (29%), while cancerous replacement of the liver accounted for most of the other patients with benign or malignant biliary obstruction identified in 5.7% [4].

Though corticosteroids are the mainstay for most high-grade immune-related adverse events such as pembrolizumab-induced hepatitis, the dose and duration of corticosteroid therapy are not well-established. Literature search revealed a case of successful management of grade 3 pembrolizumab-induced hepatitis with a combination of low-dose corticosteroids and bicyclol [5].

In addition, plasmapheresis was performed for another patient with pembrolizumab-induced myocarditis because of deterioration in his hemodynamic status refractory to conventional immunosuppression. The plasmapheresis seemed to cause a rapid reduction of serum pembrolizumab levels and marked clinical, radiological, and biochemical improvement. To the authors' knowledge, that was the first reported case on the successful use of plasma exchange for pembrolizumab-associated fulminant myocarditis [6].

Some degree of our patient's short-term improvement in biochemical parameters was likely due to direct removal via plasmapheresis. There was clear downtrend in alkaline phosphatase, AST, ALT, and total bilirubin each day after plasmapheresis followed by an uptrend or stagnant value the following day when patient did not receive plasmapheresis. However, his long-term improvement cannot be solely due to direct removal, as his recovery of liver injury was durable.

Also, some of these same laboratory markers were on a downward trend prior to plasmapheresis. Thus, it is plausible that plasmapheresis did not hasten his recovery. The total bilirubin was an exception, as it plateaued after all of our other immunosuppression therapies prior to the initiation of plasmapheresis. While alkaline phosphatase and aminotransferases were on a downward trend prior to initiation of plasmapheresis, the improvement trend increased on the day of initiating plasmapheresis and remained increased until normalization. It is plausible that the patient may have recovered on his own but at a slower rate. In other words, plasmapheresis may have increased the rate of recovery at worst and may have been an essential factor in his recovery at best. The half-life of IgG is about 23 days, so there was most likely significant amounts of pembrolizumab in the patient about 4–6 weeks after administration. Moreover, IgG is about 50% intravascular, so the rationale for some degree of drug removal via plasmapheresis seemed sound.

## Data Availability

Data come from the medical record.
